# Prediction of Clinical Outcomes in Hepatitis B E Antigen Negative Chronic Hepatitis B Patients with Elevated Hepatitis B Virus DNA Levels

**DOI:** 10.1371/journal.pone.0144777

**Published:** 2015-12-22

**Authors:** Jem Ma Ahn, Dong Hyun Sinn, Geum-Youn Gwak, Yong-Han Paik, Moon Seok Choi, Joon Hyeok Lee, Kwang Cheol Koh, Seung Woon Paik

**Affiliations:** 1 Department of Medicine, Samsung Medical Center, Sungkyunkwan University School of Medicine, Seoul, Korea; 2 Department of Medicine, Korea University School of Medicine, Seoul, Korea; National Taiwan University College of Medicine, TAIWAN

## Abstract

**Objectives:**

We investigated whether long-term clinical outcomes such as disease progression or inactive hepatitis B virus (HBV) carrier state can be predicted by baseline factors in hepatitis B e antigen (HBeAg)-negative HBV infected patients with an elevated viral load.

**Methods:**

A retrospective cohort of 527 HBeAg-negative chronic HBV infected patients with an elevated viral load (HBV DNA ≥ 2,000 IU/ml) was assessed for disease progression defined by the development of hepatocellular carcinoma (HCC) or cirrhotic complication, as well as becoming an inactive carrier.

**Results:**

During a median 3.6 years of follow-up, disease progression was detected in 46 patients (40 with HCC, 6 with cirrhotic complication), and 31 of 309 non-cirrhotic patients became inactive carriers. Older age, male gender, cirrhosis, high HBV DNA levels at baseline, and short antiviral therapy duration were independent risk factors for HCC. Low HBV DNA and quantitative hepatitis B surface antigen (qHBsAg) levels were independent predictors for becoming inactive carriers in patients without cirrhosis. In non-cirrhotic patients with both low qHBsAg and HBV DNA levels, the 5-year cumulative incidence of an inactive carrier was 39.8%, while that of disease progression was 1.6%.

**Conclusion:**

HBeAg negative patients without cirrhosis can be closely monitored for becoming an inactive carrier when both HBV DNA and qHBsAg levels are low, as the risk of disease progression is low while incidence of an inactive carrier is high.

## Introduction

Chronic hepatitis B virus (HBV) infection is a major etiology of chronic hepatitis, cirrhosis and hepatocellular carcinoma (HCC) worldwide as well as in Korea [1, 2]. However, not all patients chronically infected with HBV have a dismal prognosis. During the course of chronic HBV infection, some patients naturally enter into the inactive HBV carrier phase, which is characterized by negative hepatitis b e antigen (HBeAg), persistently normal alanine aminotransferase (ALT) levels, and HBV DNA levels < 2,000 IU/ml [1–3]. Although some of them may revert to HBeAg-negative active hepatitis [4], many patients remain in the inactive phase with a good long-term prognosis. In contrast, most patients in the HBeAg negative chronic hepatitis B (CHB) phase, which is characterized by HBeAg negativity, persistently or intermittently elevated levels of ALT, and HBV DNA levels ≥ 2,000 IU/ml, have an increased risk of cirrhosis and HCC [5].

Sometimes it can be difficult to distinguish patients in the inactive HBV-carrier phase from those in the HBeAg-negative CHB phase. Not all HBeAg negative patients with elevated HBV DNA levels are in the HBeAg-negative CHB phase, as fluctuations can occur in HBV DNA and ALT levels [1, 2]. Therefore, clinicians sometime closely monitor HBeAg negative patients with elevated HBV DNA levels to distinguish the HBeAg-negative CHB phase from the inactive HBV carrier phase. This careful monitoring strategy is required to avoid unnecessary treatment; however, there is a risk of disease progression during the careful monitoring period. In this sense, early prediction of long-term outcome in HBeAg-negative patients with an elevated HBV DNA level has paramount importance for planning the management strategy. Thus, in this study, we analyzed whether long-term clinical outcomes of HBeAg-negative CHB patients with an elevated viral load can be predicted using baseline factors. In particular, we focused on whether quantitative hepatitis B surface antigen (qHBsAg) levels can help identify long-term clinical outcomes of these patients, as qHBsAg levels provide additional information to understand the clinical course of patients with chronic HBV infection [6].

## Patients and Methods

### Study design, setting, and participants

We screened consecutive HBsAg positive patients from the database of Samsung Medical Center, Seoul, Korea between January 2008 and December 2011. We included 1,199 patients who met the following inclusion criteria: 1) age ≥ 18 years; 2) chronic HBV infection, defined by the presence of HBsAg in serum for ≥ 6 months or by clinical history; 3) HBeAg negative; 4) HBV DNA ≥ 2,000 IU/ml; 5) qHBsAg measured at the same day as HBV DNA and 6) no history of cirrhotic complication, such as variceal hemorrhage, ascites, spontaneous bacterial peritonitis, hepatorenal syndrome, or hepatic encephalopathy. These patients were further evaluated and excluded from the study if any of the following exclusion criteria were met: 1) history of HCC or other chronic liver disease (n = 389); 2) history or current use of antiviral therapy (AVT) [either nucleos(t)ide analogues (NUC) or interferon] (n = 117); 3) HCC detected within 6 months from baseline or follow-up duration ≤ 6 months (n = 166). Finally, 527 treatment-naïve HBeAg negative CHB patients were analyzed. The study was reviewed and approved by the Institutional Review Board of Samsung Medical Center (IRB No. 2014-07-032-001). Because the study is based on the retrospective analysis of existing administrative and clinical data, the requirement of obtaining informed patient consent was waived by the Institutional Review Board of Samsung Medical Center. Patient records/information was anonymized and de-identified prior to analysis.

#### Study endpoint assessments and follow up

The clinical end-points were disease progression and becoming an inactive carrier during the follow-up period. Disease progression was defined as the development of HCC or cirrhotic complication, such as variceal hemorrhage, ascites, spontaneous bacterial peritonitis, hepatorenal syndrome, or hepatic encephalopathy [1]. HCC was diagnosed either by histology or clinically according to the regional HCC guidelines [7]. An inactive carrier was defined when a non-cirrhotic patient had persistent normal levels of ALT (< 40 U/L) more than 2 times plus HBV DNA levels < 2,000 IU/mL for the duration ≥ 12 months until the last follow-up in the absence of AVT [2, 3]. Follow-up started at the time of the HBV DNA level measurement, and patients were followed up every 3–6 months with at least 6-month interval. Person-years were censored on the date the end points (disease progression or inactive carrier state) were diagnosed or the last follow-up date, whichever came first.

#### Study variables

We collected the following variables at baseline: age, gender, aspartate aminotransferase (AST), ALT, albumin, bilirubin, prothrombin time, platelet count, HBV DNA levels and qHBsAg levels. Serum qHBsAg was measured using the Architect HBsAg QT (Abbott Laboratories, Chicago, IL, USA) according to manufacturer’s instructions [8]. The detection range of qHBsAg was 0.05 to 250 IU/ml; if qHBsAg was ≥ 250 IU/ml, the sample was diluted 1:500 or more. Serum HBV DNA was quantified using a real-time PCR method (COBAS Taq Man HBV assay [Roche Diagnostics, Branchburg, NJ, USA]), with a detection range of 9–170,000,000 IU/ml. Serologic viral markers (HBsAg, anti-HBs, HBeAg, anti-HBe, and anti-hepatitis C virus) were reviewed and used to determine participant eligibility. Abdominal ultrasonography or computed tomography and upper endoscopy results at baseline (or within 6 months) were also reviewed, and used to define cirrhosis. Liver cirrhosis was defined clinically with indicators such as thrombocytopenia (< 150 x 10^3^ /L), splenomegaly (by imaging), varices (by upper endoscopy), or typical cirrhotic configuration of the liver on imaging studies (nodular liver surface or caudate lobe hypertrophy) [9, 10]. During follow up, laboratory data including AST, ALT, albumin, bilirubin, prothrombin time, platelet count, HBV DNA levels, HBeAg, and anti-HBe were collected at every visit with at least 6-month interval. Abdominal ultrasonography with and without alpha fetoprotein measurement were also performed for HCC surveillance with at least 6-month interval. Starting AVT during the follow-up was also recorded. No patient used interferon during the follow-up period, and those who started AVT used NUC. A HBV DNA level ≥ 2,000 IU/mL with an elevated ALT level (≥ 80 U/L) at a certain point usually led to the initiation of AVT, according to the pharmaceutical reimbursement policy in Korea.

#### Statistical analysis

Continuous variables are expressed as means ± standard deviation or median (ranges) and categorical variables are expressed as percentages. qHBsAg and HBV DNA levels were skewed, and were log transformed and tested. Patients were categorized into cirrhotic and non-cirrhotic groups and tested again. The Cox proportional hazards regression model was used to identify factors associated with the end-points (HCC, cirrhotic complication, or inactive carrier) and to calculate the crude and multivariate hazard ratio (HR). The multivariate HR was derived by adjusting variables with a p < 0.10 in a univariate analysis. qHBsAg was included in the multivariate model, regardless of p value, as this value was variable of interest in this study. HBV DNA levels were categorized into intermediate viral load (2,000–20,000 IU/mL) and high viral load (>20,000 IU/ml). qHBsAg levels had no known cutoff values, so we tested different cutoffs using the time-dependent area under the receiver operating characteristic curve (AUC). As the time to start AVT was not uniform, we used AVT duration (which is a time-dependent variable), instead of AVT (yes vs. no), in the Cox’s regression analyses. The cumulative incidence of the end-points was estimated by the Kaplan Meier curve analysis and the log-rank test was used to identify differences. A p < 0.05 was considered significant.

## Results

### Baseline characteristics and development of clinical endpoints

The baseline characteristics of the patients are shown in Table 1. Of the patients, 218 (41.4%) had cirrhosis at baseline. Patients with cirrhosis were older, more often male, had higher AST, ALT, and bilirubin levels, as well as prothrombin time and showed lower albumin and platelet levels than those without cirrhosis. The mean HBV DNA level was higher in the cirrhosis group than that in the non-cirrhosis group, whereas the mean qHBsAg level was lower in the cirrhosis group than that in the non-cirrhosis group.

**Table 1:** Baseline characteristics and follow-up data.

Characteristics	All (n = 527)	Cirrhosis (+) (n = 218)	Cirrhosis (-) (n = 309)	P value
**Baseline characteristics**				
Age (years)	48.8± 9.9	51.5 ± 8.7	46.8 ± 10.2	< 0.001
Male (n, %)	311 (59.0)	141 (64.7)	170 (55.0)	0.03
AST (U/L)	34 (25–54)	43 (30–63)	29 (23–47)	< 0.001
ALT (U/L)	37 (24–66)	40 (27–69)	34 (22–63)	0.01
Albumin (mg/dl)	4.4 (4.1–4.6)	4.3 (3.9–4.5)	4.5 (4.3–4.7)	< 0.001
Bilirubin (mg/dl)	0.8 (0.6–1.1)	0.9 (0.7–1.2)	0.8 (0.6–1.0)	< 0.001
PT (INR)	1.0 (1.0–1.1)	1.1 (1.0–1.2)	1.0 (1.0–1.1)	< 0.001
Platelet (x 10^3^/L)	168 (128–210)	118 (94–138)	199 (173–229)	< 0.001
HBV DNA (log_10_ IU/ml)	4.9 ± 1.2	5.1 ± 1.3	4.7 ± 1.1	<0.001
qHBsAg (log_10_ IU/ml)	3.3 ± 0.6	3.2 ± 0.6	3.4 ± 0.6	< 0.001
Follow-up data				
AVT during follow-up (n, %)	311 (59.0)	175 (80.3)	136 (44.0)	< 0.001
Disease progression (n, %)	46 (8.7)	39 (17.9)	7 (2.3)	< 0.001
HCC	40 (7.6)	33 (15.1)	7 (2.3)	< 0.001
Cirrhotic complication	6 (1.1)	6 (2.8)	0 (0)	0.01
Inactive carrier (n, %)	31 (5.9)		31 (10.0)	

Abbreviations: AST, aspartate aminotransferase; ALT, alanine aminotransferase; PT, prothrombin time; INR, international normalization ratio; HBV, hepatitis B virus; qHBsAg, quantitative hepatitis B surface antigen; AVT, antiviral therapy; HCC, hepatocellular carcinoma. Values are expressed as mean ± standard deviation, median (quartile), or number (%).

The median follow-up duration was 3.6 years (range, 0.5–5.8 years). More than half (311 patients, 59.0%) of the patients started AVT during follow up (Table 1). Disease progression developed in 46 (8.7%) patients (40 patients with HCC and 6 with cirrhotic complication) (Table 1). The 1-, 3-, and 5-year cumulative incidence of disease progression was 1.4%, 7.2%, and 10.5%, respectively. When stratified by cirrhosis status, the 5-year cumulative incidence of disease progression was 2.4% for non-cirrhotic patients and was significantly higher in cirrhotic patients (21.7% at 5-years, p < 0.001). When further classified according to age, the 5-year cumulative incidence of disease progression was 1.4%, 4.0%, 12.1% and 27.6% for non-cirrhotic patients aged < 50 years, non-cirrhotic patients aged ≥ 50 years, cirrhotic patients aged < 50 years and cirrhotic patients aged ≥ 50 years, respectively (p < 0.001).

During follow-up, 31 (10.0%) of 309 non-cirrhotic patients became inactive carrier (Table 1). The 1-, 3-, and 5-year cumulative incidence of inactive carriers was 3.6%, 8.3%, and 13.1%, respectively.

### Factors associated with clinical endpoints

Older age, male gender, high HBV DNA levels and presence of cirrhosis were independent baseline risk factors for development of HCC (Table 2). The AVT duration during follow up period was also an independent factor associated with reduced risk of HCC development. However the qHBsAg level was not associated with HCC development. Moreover, qHBsAg level was not a risk factor for HCC either in patients with or without cirrhosis (data not shown).

**Table 2:** Factors associated with development of HCC.

Characteristics	Univariate HR (95% CI)	P value	Multivariate HR (95% CI)	P value
Age (per year)	1.07 (1.03–1.10)	<0.001	1.07 (1.03–1.12)	<0.001
Male (vs. female)	1.76 (0.88–3.53)	0.10	2.27 (1.11–4.63)	0.024
ALT (U/L)	0.99 (0.99–1.00)	0.40		
HBV DNA (log_10_ IU/ml)	1.39 (1.11–1.76)	0.004	1.74 (1.33–2.29)	<0.001
qHBsAg (log_10_ IU/ml)	0.72 (0.43–1.21)	0.22	0.77 (0.42–1.43)	0.42
Cirrhosis (yes vs. no)	7.06 (3.12–15.9)	<0.001	7.03 (3.02–16.3)	<0.001
AVT duration (/year)	0.84 (0.71–1.00)	0.055	0.56 (0.45–0.69)	<0.001

Abbreviations: HCC, hepatocellular carcinoma; HR, hazard ratio; CI, confidence interval; ALT, alanine aminotransferase; HBV, hepatitis B virus; qHBsAg, quantitative hepatitis B surface antigen; AVT, antiviral therapy.

Cirrhotic complication was noticed in six patients during follow-up period, which was observed exclusively in patients with cirrhosis. There was no statistically significant factor associated with cirrhotic complication, but the event of cirrhotic complication was small (n = 6).

Table 3 shows factors associated with becoming inactive carrier state in non-cirrhotic patients. Low HBV DNA and qHBsAg levels were independent predictors associated with inactive carrier state in patients without cirrhosis, while low ALT levels were associated with inactive carrier state only in univariate analysis.

**Table 3:** Factors associated with inactive carrier state in non-cirrhotic patients.

Characteristics	Univariate HR (95% CI)	P value	Multivariate HR (95% CI)	P value
Age (per year)	1.00 (0.96–1.03)	0.89		
Male (vs. female)	0.73 (0.36–1.49)	0.39		
ALT (U/L)	0.98 (0.96–0.99)	0.023	0.99 (0.98–1.01)	0.71
HBV DNA (log_10_ IU/ml)	0.16 (0.07–0.37)	<0.001	0.13 (0.05–0.35)	<0.001
qHBsAg (log_10_ IU/ml)	0.48 (0.29–0.79)	<0.004	0.36 (0.20–0.66)	0.001

Abbreviations: HR, hazard ratio; CI, confidence interval; ALT, Alanine aminotransferase; HBV, hepatitis B virus; qHBsAg, quantitative hepatitis B surface antigen.

### Incidence of clinical endpoints in the sub-cohort

When non-cirrhotic patients were stratified according to the baseline HBV DNA levels, the 5-year cumulative incidence of an inactive carrier was 25.8%, 1.7%, 2.7% and 0% for 2,000 IU/ml ≤ HBV DNA < 20,000 IU/ml (n = 149), 20,000 IU/ml ≤ HBV DNA < 200,000 IU/ml (n = 77), 200,000 IU/ml ≤ HBV DNA < 2,000,000 IU/ml (n = 52) and HBV DNA ≥ 2,000,000 IU/ml (n = 31), respectively (p < 0.001). When stratified according to the baseline qHBsAg levels, the 5-year cumulative incidence of an inactive carrier was 20.5%, 22.6%, 2.9% and 8.5% for qHBsAg < 1,000 IU/ml (n = 67), 1,000 IU/ml ≤ qHBsAg < 2,500 IU/ml (n = 74), 2,500 IU/ml ≤ qHBsAg < 5,000 IU/ml (n = 75) and qHBsAg ≥ 5,000 IU/ml (n = 93), respectively.

**Fig 1:**
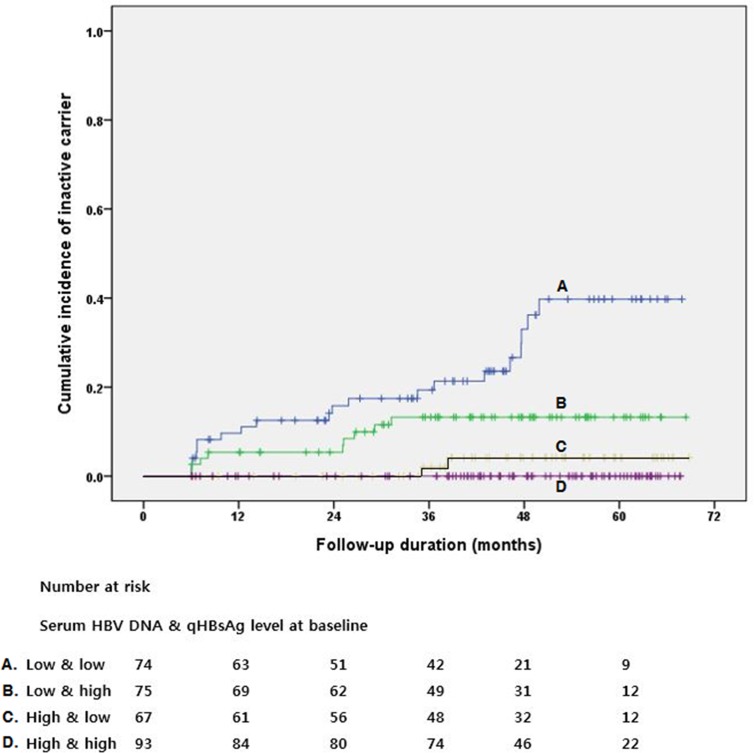
Cumulative incidence of an inactive carrier according to hepatitis B virus (HBV) DNA and quantitative hepatitis B surface antigen (qHBsAg) levels in patients without cirrhosis. Those with low qHBsAg and low HBV DNA levels showed higher cumulative incidence of an inactive carrier during follow-up period. A-D represent for low HBV DNA plus low qHBsAg levels (A), low HBV DNA plus high qHBsAg levels (B), high HBV DNA plus low qHBsAg levels (C), and high HBV DNA plus high qHBsAg levels (D), respectively. The cut-off level to define low vs. high was 20,000 IU/ml for HBV DNA and 2,500 IU/ml for qHBsAg.

Thus we categorized non-cirrhotic patients into 4 subgroups according to HBV DNA and qHBsAg levels with these cut-off values of 20,000 IU/mL for HBV DNA and 2,500 IU/ml for qHBsAg. Table 4 shows cumulative incidence of clinical endpoints in each subgroup. The 5-year cumulative incidence of an inactive carrier was 39.8%, 13.3%, 4.2% and 0% for low HBV DNA plus low qHBsAg levels, low HBV DNA plus high qHBsAg levels, high HBV DNA plus low qHBsAg levels and high HBV DNA plus high qHBsAg levels, respectively (p < 0.001) (Fig 1). Although the cumulative incidence of disease progression was low, disease progression was observed in each subgroup.

**Table 4:** Cumulative incidence of disease progression and inactive carrier state according to HBV DNA and qHBsAg levels in non-cirrhotic patients.

Groups	Disease progression			Inactive carrier		
	Person-years of follow-up	3 year	5 year	Person-years of follow-up	3 year	5 year
All (n = 309)	1144	1.4%	2.4%	1076	8.3%	13.1%
Group A (n = 74)	277	1.6%	1.6%	227	19.4%	39.8%
Group B (n = 75)	279	1.4%	1.4%	256	13.3%	13.3%
Group C (n = 67)	246	0%	4.7%	244	2.0%	4.2%
Group D (n = 93)	342	2.4%	2.4%	349	0%	0%

Abbreviation: HBV, hepatitis B Virus; qHBsAg, quantitative hepatitis B surface antigen. Group A, B, C and D are those with low HBV DNA plus low qHBsAg levels, low HBV DNA plus high qHBsAg levels, high HBV DNA plus low qHBsAg levels, and high HBV DNA plus high HBV DNA levels, respectively. Cut-off level was 20,000 IU/ml for HBV DNA and 2,500 IU/ml for qHBsAg.

These cumulative incidence rates of clinical endpoints in non-cirrhotic patients according to HBV DNA and qHBsAg levels were further sub-classified by age group (Table 5). Inactive carriers were observed in over 39.5% of young patients (< 50 years) with both low HBV DNA and low qHBsAg levels at 5-year follow up, while there was no case of disease progression in young patients with low HBV DNA levels. Older patients (≥ 50 years) also showed high cumulative incidence of inactive carrier state (42.2% at 5-year) when they had both low HBV DNA and low qHBsAg levels at baseline, while their cumulative incidence of disease progression was less than 5%.

**Table 5:** Cumulative incidence of disease progression and inactive carrier state according to HBV DNA and qHBsAg levels in non-cirrhotic patients, further stratified by age-group.

Groups	Disease progression			Inactive carrier		
	Person-years of follow-up	3 year	5 year	Person-years of follow-up	3 year	5 year
Age < 50 years (n = 180)						
Group A (n = 38)	155	0%	0%	119	25.1%	39.5%
Group B (n = 45)	172	0%	0%	163	10.1%	10.1%
Group C (n = 35)	133	4.2%	4.2%	131	3.8%	7.9%
Group D (n = 62)	238	1.8%	1.8%	241	0%	0%
Age ≥ 50 years (n = 129)						
Group A (n = 36)	121	3.6%	3.6%	108	13.1%	42.2%
Group B (n = 30)	107	3.6%	3.6%	93	17.5%	17.5%
Group C (n = 32)	113	5.6%	5.6%	113	0%	0%
Group D (n = 31)	104	3.4%	3.4%	108	0%	0%

Abbreviation: HBV, hepatitis B Virus; qHBsAg, quantitative hepatitis B surface antigen. Group A, B, C and D are those with low HBV DNA plus low qHBsAg levels, low HBV DNA plus high qHBsAg levels, high HBV DNA plus low qHBsAg levels, and high HBV DNA plus high HBV DNA levels, respectively. Cut-off level was 20,000 IU/ml for HBV DNA and 2,500 IU/ml for qHBsAg.

## Discussion

In our study, several baseline factors were independently associated with development of HCC, which were older age, male gender, high HBV DNA levels, and presence of cirrhosis. Furthermore AVT duration during follow up was significantly associated with reduced risk of HCC development. Our findings are in line with several previous studies that reported risk factors for HCC in chronic HBV infected patients [3, 5]. Guidelines for management of hepatitis B recommend prompt antiviral therapy for cirrhotic patients with an elevated viral load [2, 3]. Our data also support that cirrhotic patients should receive prompt antiviral therapy, as the risk of disease progression was substantial (21.7% at 5-years) and as the risk of HCC was reduced by increasing AVT duration. In contrast, the 5-year cumulative incidence of disease progression was low for patients without cirrhosis (2.4% at 5-years). The rate was even lower (1.4% at 5-years) for young patients (age < 50 years), while quite a large proportion of patients without cirrhosis (8.3% at 3-years and 13.1% at 5-years) became inactive carriers. Thus close monitoring is an attractive option, as currently available AVT just controls viral replication without eradication so it should be received life-long until the HBsAg disappears [5].

We found that HBV DNA and qHBsAg levels help select those who may benefit from close monitoring over prompt therapy. qHBsAg and HBV DNA levels were significant factors associated with inactive carriers. Our results were consistent with those of previous cross-sectional studies showing that the qHBsAg level is lower in inactive carrier state than in immune tolerance or immune clearance phase [11, 12], those of previous retrospective cohort studies that showed low qHBsAg levels can predict maintenance of inactive carrier states [6, 13], or those of a prospective cohort study showing that qHBsAg levels help to identify inactive carrier state from active CHB phase in genotype D HBeAg negative patients [14]. The 5-year cumulative incidence of an inactive carrier was 42.2% for non-cirrhotic patients aged ≥ 50 years with both low HBV DNA and low qHBsAg levels, and it was 39.5% for < 50 years. In contrast, the cumulative incidence of an inactive carrier was 0% at 5-years in either non-cirrhotic patients aged ≥ 50 years or those aged < 50 years when both HBV DNA and qHBsAg levels were high. Those with high HBV DNA and low qHBsAg levels or those with low HBV DNA and high qHBsAg levels were in between. These data show that HBsAg and HBV DNA levels are useful to identify patients who will become inactive carriers among those with HBeAg negative CHB and an elevated viral load, and can help select patients who may benefit from close monitoring over prompt AVT. We further assessed the risk of disease progression in non-cirrhotic patients stratified by HBV DNA levels, qHBsAg levels and age. Notably, there was no case of cirrhotic complication in non-cirrhotic patients, and all the cases were HCC. In this analysis, we could note that the risk of disease progression was generally low in non-cirrhotic patients with low qHBsAg plus low HBV DNA levels, especially when they were young (age < 50 years). This suggested that young patients with low HBV DNA (< 20,000 IU/ml) plus low qHBsAg levels (< 2500 IU/ml) may be monitored over prompt AVT.

In this study, qHBsAg levels were not related with development of HCC. Consistent with our results, another study reported that qHBsAg levels are not associated with development of HCC in patients with an elevated viral load [15]; in that study, qHBsAg levels did not predict HCC in HBeAg negative patients with HBV DNA ≥ 2,000 IU/mL (p = 0.247) [15]. In contrast, a study from Japan reported that the qHBsAg level is a significant predictor for the development of HCC in patients treated with NUC [16], suggesting qHBsAg levels may have value in prediction of HCC in patients receiving AVT. However, the data from Japan are limited by a small sample size (167 patients and nine with HCC). In our study, about 50% of patients started AVT during follow-up at a different time point for each patient, so our data were also limited in answering this clinical question. Whether qHBsAg levels may have a role predicting development of HCC in NUC treated patients is still an open question, which warrants further study.

Our study has several limitations. This was a retrospective study with inherent limitations. HBV genotype can influence incidence of HCC as well as qHBsAg loss [1, 3, 17], which we did not investigate in this study, as almost all Korean patients are infected with HBV genotype C [18]. Thus application to other HBV genotype remains to be determined. In addition, presence of pre S/S mutant, one of risk factors for HCC, affects serum qHBsAg levels [19], which we did not investigate. Fibrosis stage of the liver was also missing and cirrhosis was defined clinically by thrombocytopenia, varices, or radiologic findings. In this study, patients were treatment naïve patients, but subsequently started AVT during follow-up. AVT is a well-known factor associated with development of HCC, and yet AVT was initiated at variable time point during follow-up. We used time-dependent variables (AVT duration) instead of use of AVT (yes vs. no) to minimize potential bias. However, AVT may act as a potential bias in this study, as time to start AVT was different from person to person. Finally, the rate of clinical event was relatively low (e.g., patients with cirrhosis complications = 6) and the follow-up period was not long enough. Therefore, our data need to be interpreted in the context of these limitations.

Despite these limitations, this study was a large scale study with clinical implications. Some HBeAg negative patients with an elevated viral load showed controlled HBV replication (persistent decrease of serum HBV DNA < 2,000 IU/ml combined with normal ALT levels in the absence of AVT). These results show that not all HBeAg negative patients with elevated HBV DNA levels are in HBeAg-negative CHB state requiring prompt AVT. Cirrhotic patients should receive prompt AVT, as the risk of disease progression is substantial. However, for some of patients without cirrhosis, close monitoring can be considered, as the risk of disease progression is low while incidence of an inactive carrier can be high. Our data indicate that qHBsAg and HBV DNA levels are helpful for selecting those who may benefit from monitoring among patients without cirrhosis.

In summary, our study found that older age, male gender, high HBV DNA level, cirrhosis and short AVT duration were independently associated with development of HCC in HBeAg negative patients with elevated viral loads, while low HBV DNA and qHBsAg levels were significant predictors for becoming inactive carriers in non-cirrhotic patients. Thus our study suggested that HBeAg negative patients without cirrhosis can be closely monitored when both HBV DNA and qHBsAg levels are low, as the risk of disease progression is low while incidence of an inactive carrier is high.
